# Protective and Predisposing Morphological Factors in Suprascapular Nerve Entrapment Syndrome: A Fundamental Review Based on Recent Observations

**DOI:** 10.1155/2017/4659761

**Published:** 2017-06-13

**Authors:** Piotr Łabętowicz, Marek Synder, Mariusz Wojciechowski, Krzysztof Orczyk, Hubert Jezierski, Mirosław Topol, Michał Polguj

**Affiliations:** ^1^Department of Angiology, Interfaculty Chair of Anatomy and Histology, Medical University of Łódź, Narutowicza 60, 90-136 Łódź, Poland; ^2^Clinic of Orthopedics and Pediatric Orthopedics, Medical University of Łódź, 90-131 Łódź, Poland; ^3^Department of Orthopedy, Brüderkrankenhaus St. Josef Paderborn Clinic, University of Göttingen, Schlossplatz 2, Wilhelmsplatz 1, 37073 Göttingen, Germany; ^4^Department of Orthopedics and Traumatology, Ministry of the Interior Hospital, Północna Str. 42, 91-245 Łódź, Poland; ^5^Department of Normal and Clinical Anatomy, Interfaculty Chair of Anatomy and Histology, Medical University of Łódź, Narutowicza 60, 90-136 Łódź, Poland

## Abstract

Suprascapular nerve entrapment syndrome (SNES) is a neuropathy caused by compression of the nerve along its course. The most common compression sites include the suprascapular notch and the spinoglenoid notch. The aim of this article was to review the anatomical factors influencing the occurrence of SNES in the light of the newest reports. Potential predisposing morphological factors include a V-shaped, narrow, or “deep” suprascapular notch; a band-shaped, bifurcated, or completely ossified superior transverse scapular ligament (STSL); particular arrangements of the suprascapular nerve and vessels at the suprascapular notch. A very recent report indicates structures at the suprascapular notch region that may protect from SNES, such as the suprascapular notch veins (SNV). The role of the anterior coracoscapular ligament (ACSL) is still not clear. While some studies indicate that it may predispose for SNES, the newest study proposes a protective function. Knowledge of these variations is essential for arthroscopic and other surgical procedures of this area in order to avoid iatrogenic injury of the suprascapular nerve or unexpected bleeding from the suprascapular vessels running alongside the STSL.

## 1. Introduction

Suprascapular nerve entrapment syndrome (SNES) is a neuropathy in which the nerve is compressed along its course, most commonly at the suprascapular notch (SSN). The suprascapular nerve always passes through the SSN below the superior transverse suprascapular ligament (STSL) before entering the supraspinous fossa [1, 2]. Suprascapular neuropathy, initially described by Andre Thomas in 1936 [3], is the cause of about 1-2% [4] of conditions resulting in pain and dysfunction of the shoulder girdle. Symptoms caused by nerve compression are based on progressive atrophy of the supraspinatus and infraspinatus muscles supplied by the suprascapular nerve [5]. Traumatic injuries such as scapular fracture, clavicular fracture, proximal humerus fractures, dislocation of the shoulder, or the acromioclavicular joint are common causes of nerve damage [6–10]. Additional causes of neuropathy include iatrogenic injuries during surgical procedures, exertional overload in athletes or physical labourers, tuberous changes of this area (like ganglion cyst, bone cyst, osteosarcoma, soft tissue sarcoma, and metastatic lesions), or even systemic diseases like systemic lupus erythematosus (SLE) or rheumatoid arthritis [4, 10–16].

It is becoming more common to examine the anatomical basis of SNES. Rengachary et al. [17] postulate that neuropathy may occur due to irritation of the nerve by the sharp borders of the suprascapular notch while traversing from the anterior site of the scapula to the supraspinatus fossa, this situation being referred to as the “sling effect.” Sandow and Hie [13] note that it is common for athletes to compress the infraspinatus branch of the suprascapular nerve on excessive abduction with the full rotation of the arm. Ringel et al. [18] suggest that damage to the intima in the suprascapular artery caused by its outstretching and the formation of microemboli obstructing the vessel may trigger the symptoms of SNES.

The diagnosis of SNES is typically based on interview, physical examination, and additional tests [4, 19, 20]. The diagnosis should be differentiated from damage to the brachial plexus, diseases of the cervical part of the spinal cord, cervical discopathy, or diseases of the shoulder joint, for example, degeneration of the shoulder or damage to the rotator cuff [9, 13]. Other possible tests include medical imaging (X-ray, ultrasonography, MRI, and CT) [21, 22] and electrodiagnostic study (electromyography) and evaluation of the conduction speed from the nerve point of the neck to the supra- and infraspinatus muscles [14, 19].

## 2. Morphological Factors Predisposing to SNES

Potential anatomical reasons which predispose a patient to SNES include the shape of the suprascapular notch [23]; band-shaped [24], bifurcated [25], or completely ossified [26] STSL; the presence of the anterior coracoscapular ligament [1] or spinoglenoid ligament [25, 27]; the course of the suprascapular nerve and vessels [28]; the structural type of the inferior transverse scapular ligament (ITSL) [29, 30]; hypertrophy of the infraspinatus muscle [31].

### 2.1. Shape of Suprascapular Notch (SSN)

There are several classifications of the shape of the suprascapular notch (Table 1). The first classification created by Hrdicka [32] distinguished five types of SSN based on its general appearance: whether it was absent (I), shallow (II), medium (III), or deep (IV) or formed a complete foramen (V). An important modification introduced by Rengachary et al. [33] accounted for the fact that SSN may be U- or V-shaped. Based on this revised classification, Type IV, the variant that includes the narrowest V-shaped notch, is the main variant predisposing to SNES [34, 35].

The first classification which not only is based on a qualitative assessment of the appearance of the SSN but also took into account its actual dimensions was described by Polguj et al. [36]. The discriminating criterion was defined as the difference between the maximum depth (MD) and the superior transverse diameter (STD) of the SSN. The value of this classification has since been confirmed clinically in computed tomography [37, 38] and in ultrasonographic studies [39].

**Figure 1:**
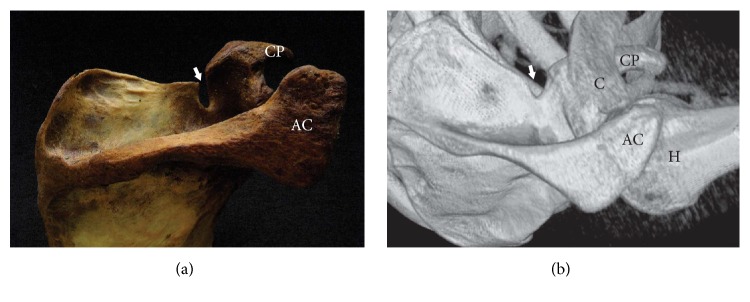
Deep and narrow suprascapular notch (arrow). (a) The dry scapula and (b) three-dimensional volume rendering MDCT. AC: acromion, C: clavicle, CP: coracoid process, and H: humerus.

The shape of the SSN is strongly dependent on the sex of the patient [40]. The deep and narrow SSN is more common in men (28.45%) than in women (18.66%) while the broad and shallow SSN is more frequent in women (63.06%) than in men (50.87%) [37]. According to previous studies, males are three to four times more likely to suffer from suprascapular neuropathy [4, 21, 35, 41, 42], which is supported by the “sling effect” hypothesis [33] described above. Therefore, the “deep” (Figure 1(a)) and narrow V-shaped suprascapular notch is the most likely to induce injury by irritation (Figure 1(b)). This is especially important for baseball pitchers, volleyball players, and tennis players [43, 44]. The frequency of this pathology in international level high-performance volleyball players was 33% [44].

### 2.2. Types of Superior Transverse Scapular Ligament (STSL)

The STSL connects the two borders of the SSN, closing them into the foramen [45, 46]. The shape of STSL is highly variable: it may also even consist of two or three parts as a bifid [31, 34, 45, 46] or trifid ligament [45, 47]. There are two classifications of STSL based on distinct parameters (Table 2).

Bayramoglu et al. [34] classify the STSL on a qualitative basis using only morphological observation. They describe five types of STSL, with ossified ligament classed in a separate category. They define Type I as uniform and fan-shaped (53.1%), Type II as fan-shaped but with the additional presence of an ACSL (18.8%), Type III as having anterior and posterior parts (15.6%), and Type IV as being calcified (12.5%).

Polguj et al. [24] distinguish only three types of STSL on the basis of morphology and measurement of the ligaments. Based on their classification, the STSL may appear as a fan-shaped ligament (Type I), defined as having a proximal width at least twice that of its distal width (54.6%). Type II, with band-shaped structures, was defined as having a proximal to distal width ratio of less than two (41.9%). Type III was defined as bifid (3.5%) [24].

**Figure 2:**
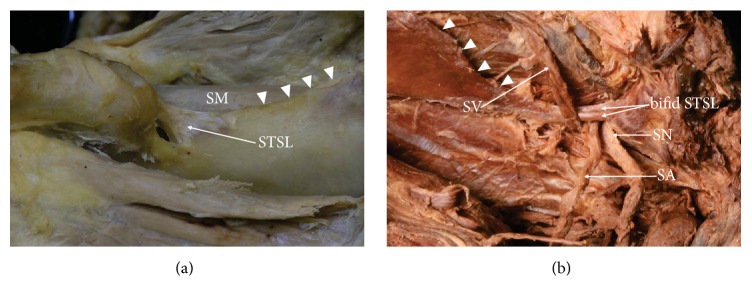
Formalin-fixed cadaveric shoulders: suprascapular region. (a) Band-shaped STSL and (b) bifid STSL. Arrowhead: superior border of the scapula, SA: suprascapular artery, SN: suprascapular nerve, SM: supraspinatus muscle, STSL: superior transverse scapular ligament, and SV: suprascapular vein.

Quantitative analysis revealed significant differences between the specimens with fan-shaped and band-shaped types of STSL in the area of the suprascapular opening. This parameter was smaller in the band-shaped STSL, and so this type may be associated with a greater chance of suprascapular nerve entrapment syndrome (Figure 2(a)).

Only one classification of bifid STSL exists in the literature [25]. It describes one subtype where the STSL is split frontally (upper and lower bands) and a second subtype where the ligament is split in the transverse plane (anterior and posterior bands). In the former (Subtype I), the bifurcated end of the ligament attaches along the medial edge of SSN, whereas, in Subtype II, two bands attach along the lateral edge of SSN [25]. The study also notes that the mean area of the suprascapular opening for suprascapular nerve passage in the specimens with singular STSL was larger than in the bifid STSL (Figure 2(b)).

### 2.3. Completely Ossified Superior Transverse Scapular Ligament (STSL)

**Figure 3:**
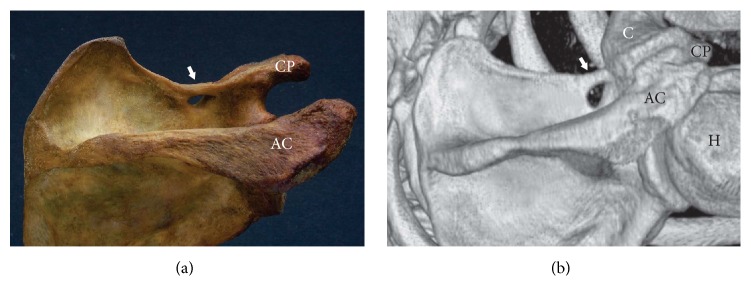
Completely ossified superior transverse scapular ligament (arrow). (a) The dry scapula and (b) three-dimensional volume rendering MDCT. AC: acromion, C: clavicle, CP: coracoid process, and H: humerus.

A completely ossified STSL is one of the most important predisposing factors for suprascapular nerve entrapment syndrome [26, 33, 36, 48] (Figure 3). It occurs more often in men (6.4%) than in women (3.75%) and the difference is statistically significant (*p* = 0.01537) [38]. This difference may account for the greater prevalence of suprascapular neuropathy in men. The presence of an ossified STSL varies between regions worldwide [38] (Table 3). In Europeans, it varies from 1.5% in Finland [49] to 12.5% in Turkey [50], whereas different studies have reported occurrences ranging from 0.3% to 6.34% in the United States [1, 23, 32, 33, 45, 51–54]. In small isolated populations, including Alaskan Eskimos or Native Americans, the frequency is as low as 0.3% [32] and 2.1–2.9% [50], respectively. Ossification of the STSL may lead to the shortening of the ligament [55]. An ossified STSL may compress the suprascapular nerve, which represents a risk factor for surgical examination of the area [33, 45].

### 2.4. Anterior Coracoscapular Ligament (ACSL)

The anterior coracoscapular ligament (ACSL) was initially described in 2002 [1]. Avery et al. [1], followed by Bayramoglu et al. [34], postulated its presence as another factor contributing to the neuropathy of the suprascapular nerve.

**Figure 4:**
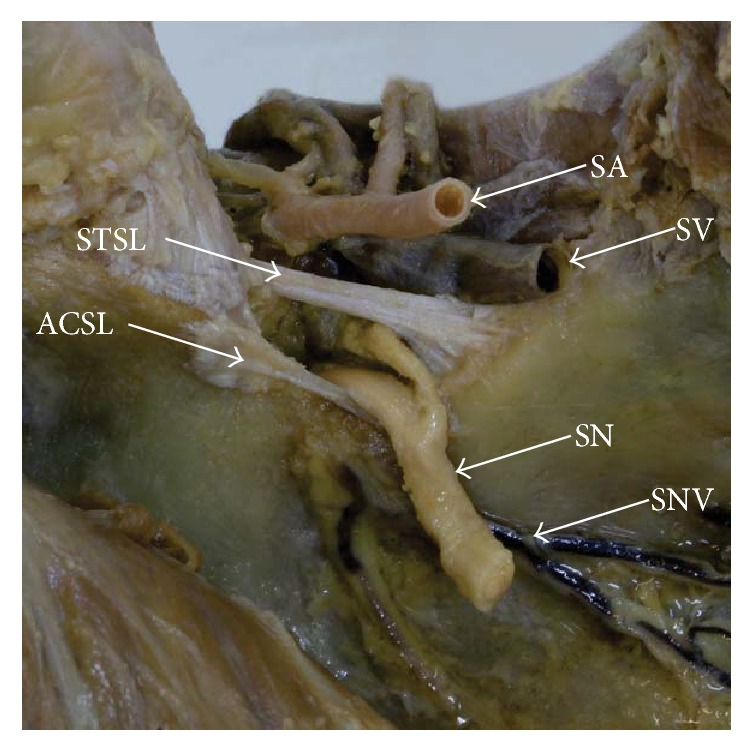
Formalin-fixed cadaveric shoulders: suprascapular region. ACSL: anterior coracoscapular ligament, SA: suprascapular artery, SN: suprascapular nerve, SNV: suprascapular notch vein, STSL: superior transverse scapular ligament, and SV: suprascapular vein.

The ACSL attaches proximally to the anteromedial surface of the root of the coracoid process. The ligament is located anteriorly to the suprascapular foramen and below the STSL (Figure 4). Histologically, the ACSL is composed of a bunch of collagen fibres with a regular pattern [1].

Avery et al. [1] and Bayramoglu et al. [34] suggest that the ACSL reduces the space for the suprascapular nerve under the STSL. However, these results were based only on macroscopic observations. The first study to evaluate the space under STSL available for the course of the suprascapular nerve, performed by Polguj et al. [24], did not find any statistically significant difference between cadavers with and without ACSL. The same authors presented the first quantitative classification of ACSL based on measurements of the ligament in 2012 [64]. Four types were distinguished (Table 4).

Also in 2012, Piyawinijwong and Tantipoon proposed a threefold classification of the ACSL based only on macroscopic observations [28]. In Type I, the distal attachment is extended to the anterior surface of scapula and continues on to the edge of the SSN. In Type II, the distal end of the ligament passes through the suprascapular foramen and runs to the other edge of the SSN, dividing the notch into two parts. In Type III, distal attachment is located near the bottom of the SSN. Type II occurs most frequently (63.16%), and this is the variant which mostly predisposes to SNES, as it reduces the depth of the suprascapular foramen [28].

The first description of a bifid ACSL was provided by Polguj et al. [64]. Bifid ligament had a uniform lateral end attached to the lateral edge of the SSN, whereas the medial end formed two bands separately attached to the medial edge of the notch. In the case given by Polguj et al. [67] the suprascapular vein ran over the ACSL, but the suprascapular artery and nerve ran below. In this position, the nerve was in contact with the bone forming the inferior border of the SSN, which increases the likelihood of SNES. In contrast to the aforementioned variant, the suprascapular nerve may run beneath the entire ACSL [68]. Such a configuration results in a higher risk for SNES [68]. This awareness is very important for Bankart arthroscopy, as well as other procedures in this area [50, 68, 69].

Gürses et al. [70] did not report any case of bifid ACSL in a Turkish population. They report the presence of an STSL in all cadavers, whereas an ACSL was found in 32% of cases. The suprascapular nerve ran between the STSL and ACSL whenever the latter was present.

### 2.5. Course of Suprascapular Nerve and Vessels

**Figure 5:**
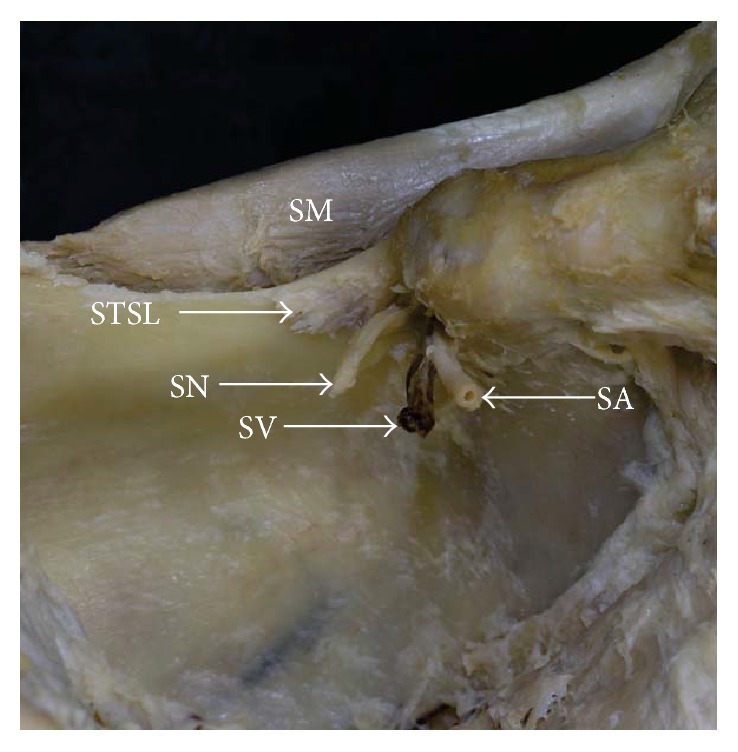
Formalin-fixed cadaveric shoulders: suprascapular region. SA: suprascapular artery, SM: supraspinatus muscle, SN: suprascapular nerve, STSL: superior transverse scapular ligament, and SV: suprascapular vein.

Arrangements of the suprascapular triad represented another important factor in the pathomechanism of SNES. The suprascapular nerve always runs under the STSL [24, 33, 59]. The course of the suprascapular vessels is highly variable [59, 60, 65] (Table 5). Yang et al. [65] identify three main variants. In Type I, both the artery and vein extend over the ligament. Type II has four subtypes, in all of which the suprascapular vessels run both above and below the ligament. In contrast, Polguj et al. [66] distinguish four types of the arrangement of the suprascapular nerve, vein, and artery at suprascapular notch, based on their course. In Type III, the entire suprascapular triad runs below the ligament, resulting in the most significant reduction of the space beneath the STSL of all the distinguished types (Figure 5). Their observations were supported by those of other authors, who indicate that, based on macroscopical observations, Type III is the most likely to induce SNES [28, 34, 71].

Tubbs et al. [52] report that whenever the suprascapular artery ran together with the suprascapular nerve under the STSL, it resulted in compression of the nerve due to blood pressure. Ringel et al. [18] postulated that damage to the intima in the suprascapular artery leads to extension of the artery, resulting in microinjuries of small vessels supplying the nerve and, therefore, in progressive ischemic atrophy of the suprascapular nerve. The frequency of variations when the suprascapular artery runs below STSL in the neighbourhood of the nerve has been estimated to range from 2.5% [60] to 3.3% [72] of cases. Chen and Adds [73] also describe a case of a doubled suprascapular artery.

Variability of the suprascapular vein usually concerns not the course itself, but the number of veins [74]. In 19.4% of cases, two suprascapular veins are present, and in 1.9% there are three [65].

According to Gürses et al. [70], course of the suprascapular nerve is one of the most important risk factors for SNES. Undeniably, the pathway of the suprascapular nerve below the anterior coracoscapular ligament is a risk factor for SNSE. In such a situation, the SN is compressed, with a flat shape and a width almost twice that of normal [64]. In addition, the presence of a suprascapular nerve running between the two bands of STSL is a significantly greater risk factor for SNES than that of a single-band STSL [45].

### 2.6. Spinoglenoid Ligament (Inferior Transverse Scapular Ligament)

The spinoglenoid region is the second most common site for compression of the suprascapular nerve. Therefore, variations in the morphology of the inferior transverse scapular ligament (ITSL) may represent another risk factor for SNES. In studies, the incidence of the spinoglenoid ligament has been reported to range from 16% to 100% [25, 75]. This ligament, when present, participates in the formation of a fibro-osseous tunnel which contains motor branch of the SN supplying the infraspinatus muscle [25, 75]. As the branches are compressed in the tunnel, they may produce symptoms of SNES. In 2014, Won et al. [76] classified the spinoglenoid ligament by its shape. They distinguished three types of ligament: band-like (I), triangular (II), and irregular (III) [76]. The first type, labelled as a thin band, was previously observed by Cummins et al. [77] and Demaio et al. [78]. Accordingly, Plancher et al. [79] described irregular and quadrangular types of ligament. The triangular ITSL was originally proposed by Won et al. [76]. Moreover, the suprascapular nerve runs along the lateral margin of the scapular spine [76].

### 2.7. Coexistence of Different Risk Factors

In our opinion, the influence of morphological variations is complex and SNES formation depends on several factors. Sometimes the coexistence of such predisposing factors may increase the possibility of this pathology developing. Undeniably, both compression of the SN by the ACSL and the neighbourhood of the SA are dangerous [64]. In addition, the coexistence of a specific arrangement of the suprascapular triad with the passage of suprascapular vessels through the suprascapular notch alongside a nerve and a bifid STSL may increase the probability of developing SNES (Figure 2(b)).

## 3. Morphological Protective Factors for SNES

Apart from the aforementioned factors increasing the chance of SNES, a few conditions may prevent its development. These include the presence of an anterior coracoscapular ligament (ACSL) [67] and suprascapular notch veins [80].

### 3.1. Anterior Coracoscapular Ligament (ACSL)

Despite being considered as a factor predisposing to SNES [1, 34], high frequency of the presence of an ACSL does not correspond with a low incidence of neuropathy. Polguj et al. [64, 67] postulate that the ACSL may prevent the development of SNES unless it does not significantly reduce the space under the STSL. Such a situation can be observed when the suprascapular nerve runs over the ACSL [67], in which case the ligament acts as a support for the nerve to protect it against excessive movement and forms a flume to enable direct passage of the nerve from the front side of the scapula to the supraspinatus fossa (Figure 4). This hypothesis of the protective role of ACSL was supported by Podgórski et al. [80], who postulate that the ACSL is more common in the deep type of suprascapular notch, which is associated with a greater chance of SNES [72]. The presence of the ACSL beneath the nerve prevents irritation of the nerve by the bony border of the SSN. ACSL only plays a protective role in Types I–III, which serve the actual mechanical function [72].

### 3.2. Suprascapular Notch Veins (SNV)

The components of the suprascapular triad are not the only structures running through the SSN. The suprascapular notch veins (SNV) can be also found in this area (Figure 4). They were first described in 2014 by Podgórski et al. [80]. The SNV are not only a variant of the suprascapular vein but a separate anatomical structure [72]. They run from the front of the scapula to the back along the inferior border of the suprascapular notch, crossing the SSN in the opposite direction to the suprascapular vein [80]. Their presence does not decrease the space available for the passage of the SN below the STSL. Also, the presence of the SNV correlates with the presence of ACSL: they were found to accompany one another in 58.2% of cases [72]. Both the ACSL and the SNV are believed to be involved in the mechanical amortisation of the suprascapular nerve [81]. By running along the bottom of the SSN, the ACSL and SNV protect the suprascapular nerve from irritation by the bone borders of the notch. They play the same role when the nerve runs above ACSL [81].

### 3.3. Completely Ossified Superior Transverse Scapular Ligament (STSL)

Despite being considered as a factor predisposing to SNES [26, 33, 36, 48], high frequency of the presence of completely ossified superior transverse scapular ligament in Brazilian population (30.76%) [59] does not correspond with a higher incidence of suprascapular neuropathy in this region. Maybe an ossified ligament is thought to protect the nerve from the sling effect by the ligament. However, bony bridges are seen more in often older age suggesting that they may be related to enthesopathic changes [82, 83]. According to Rengachary et al. [17, 33], presence of ossified STSL (complete or partial) may be associated with a predilection to a traction-type injury of the suprascapular nerve. Also presence of rare anatomical variations including coexistence of the suprascapular notch with the suprascapular foramen [84] or the double suprascapular foramen [85] confirmed such hypothesis.

## 4. Clinical Factors Predisposing to SNES

Suprascapular neuropathy can result from traction injury on the nerve formed by a retracted superior or posterior rotator cuff tears [86]. As a rotator cuff retracts, the course of the suprascapular nerve is altered. Such change can cause a traction injury of the nerve [87, 88]. According to Albritton et al. [89], increasing retraction of the supraspinatus tendon led to a reduction in the angle between the suprascapular nerve and its first motor branch and thus increased tension may result in neuropathy. Mallon et al. [90] described reinnervation potentials after partial arthroscopic rotator cuff repair in patients with suprascapular neuropathy as a result of massive retracted rotator cuff tear. Also Costouros et al. [91] reported that patients with preoperative electrodiagnostically confirmed suprascapular neuropathy showed nerve recovery after partial or complete rotator cuff repair.

Besides retracted rotator cuff tears, there can be other causes of suprascapular neuropathy. One of the most common conditions resulting in compression of the suprascapular nerve is a paralabral cyst. A cyst develops as a consequence of a labral injury can compress the suprascapular nerve, typically at the spinoglenoid notch [92, 93]. However if the cyst becomes sufficiently large, the nerve can also be compressed at the suprascapular notch [90, 94].

## 5. Conclusion

The vast range of morphological variation demonstrated by structures in the area of the suprascapular notch has been examined during studies on the pathogenesis of suprascapular nerve entrapment syndrome. Based on descriptions of new anatomical structures (namely, the ACSL and SNV), several factors predisposing to and protecting from SNES can be distinguished. The pathogenesis of SNES remains complex and multifactorial. An awareness of the variation of the structures in the suprascapular notch region, particularly the mutual relations between vessels and the suprascapular nerve, is very important because surgical approaches must be carried out with caution to avoid iatrogenic damage of the suprascapular nerve or bleeding from the suprascapular vessels above and below the ligament.

## Figures and Tables

**Table 1:** Classifications of the shape of the suprascapular notch (SSN).

Year	Authors	Number of types	Type of classification
1942	Hrdicka [32]	5	Qualitative
1960	Olivier [56]	5	Qualitative
1979	Rengachary et al. [33]	6	Qualitative
1998	Ticker et al. [45]	2	Qualitative
2003	Bayramoglu et al. [34]	2	Qualitative
2007	Natsis et al. [57]	5	Qualitative
2010	Duparc et al. [31]	2	Qualitative
2010	Iqbal et al. [58]	3	Qualitative
2011	Polguj et al. [36]	5	Quantitative

**Table 2:** Classification of superior transverse scapular ligament (STSL).

Year	Authors	Number of types	Type of classification
2003	Bayramoglu et al. [34]	5	Qualitative
2012	Polguj et al. [24]	3	Quantitative

**Table 3:** Frequency of ossifications of the superior transverse scapular ligament in different populations (STSL).

Country or nation	Ossification of STSL (%)
Brazil [59]	30.76
Ancient Egyptians [32]	13.6
Turkey [34, 50]	3.0–12.5^*∗*^
Germany [57]	7.3
Poland [36, 37]	4.72–7.0^*∗*^
France [60]	6.5
United States [1, 23, 32, 33, 45, 51–54]	0.3–6.34^*∗*^
Italy [61]	6.1
China [62]	4.08
Kenya [63]	3
Native Americans [50]	2.1–2.9^*∗*^
Finland [49]	1.5
Alaskan Eskimos [32]	0.3

^*∗*^The table is sorted based on the highest incidence reported from each country.

**Table 4:** Types of anterior coracoscapular ligament (ACSL) based on Polguj et al. [64].

Type number	Descriptive name
I	Fan-shaped
II	Band-shaped
III	Bifid
IV	Residual

**Table 5:** Classification of the course of suprascapular vessels.

Year	Authors	Number of types	Type of classification
2011	Yang et al. [65]	3	Qualitative
2015	Polguj et al. [66]	4	Quantitative
